# The potential impact of COVID-19 in refugee camps in Bangladesh and beyond:  A modeling study

**DOI:** 10.1371/journal.pmed.1003144

**Published:** 2020-06-16

**Authors:** Shaun Truelove, Orit Abrahim, Chiara Altare, Stephen A. Lauer, Krya H. Grantz, Andrew S. Azman, Paul Spiegel

**Affiliations:** 1 Johns Hopkins Bloomberg School of Public Health, Baltimore, Maryland, United States of America; 2 Infectious Disease Dynamics Group, Baltimore, Maryland, United States of America; 3 Center for Humanitarian Health, Baltimore, Maryland, United States of America; 4 International Vaccine Access Center, Baltimore, Maryland, United States of America; University of Southern California Health Sciences Center, UNITED STATES

## Abstract

**Background:**

COVID-19 could have even more dire consequences in refugees camps than in general populations. Bangladesh has confirmed COVID-19 cases and hosts almost 1 million Rohingya refugees from Myanmar, with 600,000 concentrated in the Kutupalong-Balukhali Expansion Site (mean age, 21 years; standard deviation [SD], 18 years; 52% female). Projections of the potential COVID-19 burden, epidemic speed, and healthcare needs in such settings are critical for preparedness planning.

**Methods and findings:**

To explore the potential impact of the introduction of severe acute respiratory syndrome coronavirus 2 (SARS-CoV-2) in the Kutupalong-Balukhali Expansion Site, we used a stochastic Susceptible Exposed Infectious Recovered (SEIR) transmission model with parameters derived from emerging literature and age as the primary determinant of infection severity. We considered three scenarios with different assumptions about the transmission potential of SARS-CoV-2. From the simulated infections, we estimated hospitalizations, deaths, and healthcare needs expected, age-adjusted for the Kutupalong-Balukhali Expansion Site age distribution. Our findings suggest that a large-scale outbreak is likely after a single introduction of the virus into the camp, with 61%–92% of simulations leading to at least 1,000 people infected across scenarios. On average, in the first 30 days of the outbreak, we expect 18 (95% prediction interval [PI], 2–65), 54 (95% PI, 3–223), and 370 (95% PI, 4–1,850) people infected in the low, moderate, and high transmission scenarios, respectively. These reach 421,500 (95% PI, 376,300–463,500), 546,800 (95% PI, 499,300–567,000), and 589,800 (95% PI, 578,800–595,600) people infected in 12 months, respectively. Hospitalization needs exceeded the existing hospitalization capacity of 340 beds after 55–136 days, between the low and high transmission scenarios. We estimate 2,040 (95% PI, 1,660–2,500), 2,650 (95% PI, 2,030–3,380), and 2,880 (95% PI, 2,090–3,830) deaths in the low, moderate, and high transmission scenarios, respectively. Due to limited data at the time of analyses, we assumed that age was the primary determinant of infection severity and hospitalization. We expect that comorbidities, limited hospitalization, and intensive care capacity may increase this risk; thus, we may be underestimating the potential burden.

**Conclusions:**

Our findings suggest that a COVID-19 epidemic in a refugee settlement may have profound consequences, requiring large increases in healthcare capacity and infrastructure that may exceed what is currently feasible in these settings. Detailed and realistic planning for the worst case in Kutupalong-Balukhali and all refugee camps worldwide must begin now. Plans should consider novel and radical strategies to reduce infectious contacts and fill health worker gaps while recognizing that refugees may not have access to national health systems.

## Abstract 2

Paul Spiegel and colleagues estimate the potential consequences of COVID-19 outbreaks at refugee camps.

## Author summary

**Why was this study done?:**

- Forcibly displaced populations, especially those who reside in settlements with high density, poor access to water and sanitation, and limited health services, are especially vulnerable to COVID-19.
- Bangladesh, which has confirmed COVID-19 cases, hosts almost 900,000 Rohingya refugees from Myanmar in the Cox’s Bazar district, approximately 600,000 of whom are concentrated in the Kutupalong-Balukhali Expansion Site.
- The capacity to meet the existing health needs of this population is limited; an outbreak of COVID-19 within this population threatens to severely disrupt an already fragile situation.
- We conducted this study to estimate the number of people infected, hospitalizations, and deaths that might occur in the Kutupalong-Balukhali Expansion Site to inform ongoing preparedness and response activities by the Bangladesh government, the United Nations agencies, and other national and international actors.

**What did the researchers do and find?:**

- Using a dynamic model of severe acute respiratory syndrome coronavirus 2 (SARS-CoV-2) transmission, we simulated how a COVID-19 outbreak could spread within the expansion site according to three possible transmission scenarios (high, moderate, and low).
- Our results suggest that a large-scale outbreak is very likely in this setting after a single infectious person enters the camp, with 0.5%–91% of the population expected to be infected within the first three months and over 70%–98% during the first year, depending on the transmission scenario, should no effective interventions be put into place.
- Hospitalization needs may exceed the existing hospitalization capacity of 340 beds 55–136 days after introduction.

**What do these findings mean?:**

- A COVID-19 epidemic in a high–population density refugee settlement may have profound consequences, requiring increases in healthcare capacity and infrastructure that exceed what is feasible in this setting.
- As many of the approaches used to prevent and respond to COVID-19 in the most affected areas so far will not be practical in humanitarian settings, novel and untested strategies to protect the most vulnerable population groups should be considered, as well as innovative solutions to fill health workforce gaps.

## Introduction

Severe acute respiratory syndrome coronavirus 2 (SARS-CoV-2) is responsible for hundreds of thousands of confirmed cases of COVID-19, globally, to date. With growing concern about the global inability to control its spread, rapid preparation and planning are critical, particularly where this virus may have the greatest impact. More than 7.2 million people live in refugee camps and settlements worldwide, where high population density, limited water and sanitation infrastructure, and limited healthcare resources can create ideal conditions for the spread of infectious diseases.

Over 900,000 Rohingya refugees have sought refuge in Cox’s Bazar, Bangladesh; the majority arrived after 2017 fleeing ongoing violence in Myanmar (S1 Fig) [1]. Extending on flood-prone hilly terrain, the Kutupalong-Balukhali Expansion Site has 23 congested settlements with nearly 600,000 persons [2]. With over 46,000 persons per square kilometer, this site could qualify as one of the densest cities on earth [3]. The majority of refugee families share their shelters, often composed of one single room. Half of the households have access to sufficient water to meet their needs [4]; all water and hygiene facilities are public and overcrowded [5]. The refugees are at high risk for epidemics given the high population density, poor health status, and limited health services. With confirmed COVID-19 cases in Bangladesh due to local transmission and ongoing transmission in neighboring countries, the introduction of the virus into the expansion site is likely.

The health status of refugees has improved since 2017 but remains fragile. The latest crude and child mortality rates are below emergency levels, while global acute malnutrition remains high [6]. The site is served by five hospitals run by nongovernmental organizations (NGOs) and foreign governments, with a total of 340 hospital beds (5.7 beds per 10,000 population) and up to 630 hospital beds when needed (10.6 per 10,000 population). There are 24 primary healthcare centers (PHCs; 1 PHC/25,000 persons) with numerous health posts, although the total number of functioning PHCs varies [7, 8]. Outside of the refugee site, there are 910 beds available in Cox’s Bazar district, including government, private, and NGO facilities for both the host community and refugees [9, 10]. The district hospital, with a 250-bed capacity, typically treats between 400 and 600 inpatients daily, 50–60 of whom are estimated to be refugees [10, 11]. It suffers from overcrowding, with a bed occupancy rate over 200%, poor infection control, and inadequate hygiene protocol and waste management [10, 11]. While it has six intensive care unit (ICU) beds available, the ICU is reportedly not functional due to lack of trained staff and functional equipment [12]. However, the health cluster and its partners are planning to expand the health system capacity, including increasing beds and staffing. Currently, there are an estimated 0.31 physicians and 0.12 nurses per 1,000 population in Bangladesh, far below the 4.5 skilled health workers per 1,000 population recommended by the World Health Organization (WHO) [13, 14].

In this study, we aim to understand how SARS-CoV-2 might impact refugee camp populations, using the Rohingya refugees living in the Kutupalong-Balukhali Expansion Site as a case study. The primary aims of this analysis are to (1) develop a baseline expectation of the possible infection burden, speed, and hospitalization capacity needed to respond to a COVID-19 epidemic; (2) use these findings to provide some recommendations to support ongoing preparedness planning by the Bangladesh government, United Nations agencies, and other actors for a COVID-19 outbreak; and (3) apply lessons from this case study to refugees and other forcibly displaced persons globally.

## Methods

We used a stochastic Susceptible Exposed Infectious Recovered (SEIR) mathematical model to simulate transmission in this population [15]. To capture the potential variability of transmission possible in this setting, we simulated epidemics under three potential scenarios with different values of the basic reproductive number, *R*_*0*_: (1) a low transmission scenario based on transmission levels in many of the Chinese provinces with elevated isolation and control practices and an *R*_*0*_ similar to influenza (*R*_*0*_ = 1.5–2.0) [16, 17]; (2) a moderate transmission scenario that mirrors estimates in early stages of the outbreak in Wuhan, China (*R*_*0*_ = 2.0–3.0) [18]; and (3) a high transmission scenario where we assume that *R*_*0*_ is increased by a factor of 1.65 (*R*_*0*_ = 3.3–5.0) compared to estimates from other settings, as was observed during the 2017 diphtheria outbreak [19]. The *R*_*0*_ in each of these scenarios falls within the 95% confidence interval (CI) of the current range of estimates for COVID-19 [20]. We assumed an Erlang-distributed serial interval (time between the onset of symptoms in infector–infectee pairs) with a mean of 6 days (standard deviation [SD] = 4.2) [21]. We assumed a population of 600,000 individuals and that the population was essentially closed (i.e., no movement, births, or deaths other than from COVID-19). Population characteristics and parameters used are detailed in Table 1, and further details about the model are in **S1 Text**.

**Table 1:** Population characteristics and parameter values used in the model to estimate the transmission and impact of SARS-CoV-2 transmission in the Kutupalong-Balukhali Expansion Site.

Parameter	Value (range)	Source
***Population***		
Size	600,000	UNHCR 2019 [2]
Median age	16 years	UN WPP 2019[22]
Proportion female	*not accounted for*	-
***Transmission***		
Basic reproductive number, *R*_*0*_		
*Low transmission*	1.5–2.0	White et al. 2009 [17]
*Moderate transmission*	2.0–3.0	Kucharski et al. 2020 [18]
*High transmission*	3.3–5.0	Truelove et al. 2019 [19]
Mean incubation period	5.2 days	Lauer et al. 2020 [23]
Infectious period	6 days (SD = 4.2)	Bi et al. 2020 [21]
***Hospitalization and death***		
Proportion of infections resulting in hospitalization	0.036 (95% CI, 0.012–0.093)	*estimated*^1^
Proportion of hospitalizations resulting in ICU admission	0.32	Huang et al. 2020 [24]
Proportion of hospitalization resulting in death	0.10	Huang et al. 2020 [24]
Time from onset to hospitalization	median: 3.42 days (SD: 0.79 days)	Bi et al. 2020 [21]
Time from hospitalization to discharge	11.5 days (95% CI, 8.0–17.3)	Sanche et al. 2020 [16]
Time from hospitalization to ICU admission	median: 8.25 days (IQR, 4.8–14.0)	Zhang et al. 2020 [25]
Time from hospitalization to death	11.2 days (95% CI, 8.7–14.9)	Sanche et al. 2020 [16]
Birth rate	0	-

^1^see **S1 Text** for details.

Abbreviations: CI, confidence interval; ICU, intensive care unit; SARS-CoV-2, severe acute respiratory syndrome coronavirus 2; SD, standard deviation; UNHCR, United Nations High Commissioner for Refugees; WPP, World Population Prospects

In addition to the population size and potential for elevated transmission (scenario 3), we also accounted for the age of this population in our estimates of severity and death, following consistent findings that age is a primary indication for severity and death. We estimated the probability that an infection results in severe disease with a logistic generalized addative model with a penalized cubic spline for age and a random effect for study, using data from five studies[21, 26–29]. We aggregated predictions from this model into estimates for 10-year age groups and then applied those estimates to the age distribution of Kutupalong-Balukhali to get an overall probability that an infection that will develop into severe disease in this population (see **S1 Text** for more details). However, it should be noted that our estimates of severe disease and death do not account for differences in comorbidities, differing attack rates by age due to population mixing characteristics, or various other factors.

We assume that, in this setting, hospitalization would be limited to those with severe disease (defined as tachypnea [≥30 breaths/minute] or oxygen saturation ≤93% at rest, or PaO2/FIO2 <300, and/or lung infiltrates >50% of the lung field within 24–48 hours) [30] and not used as a means of isolation. Thus, we assumed the proportion hospitalized was equivalent to the age-adjusted severe disease proportion calculated for the population and applied this to incident infections from the model simulations [21]. We assumed 32% of severe cases would require intensive care [24]. We estimated deaths assuming a 10% case fatality risk rate among hospitalizations/severe disease, as was observed for SARS [31]. We conservatively did not account for potential increases in mortality when healthcare resources are exhausted. We assumed hospitalization occurs a median of 3.42 days after symptom onset (lognormally distributed, SD = 0.79), hospitalized cases are discharged after a mean of 11.5 days (95% CI, 8.0–17.3), and deaths occur after a mean of 11.2 days (95% CI, 8.7–14.9) [16].

## Results

Our results suggest that a large-scale outbreak is likely in this population, even under the low transmission scenario, with 61% of the 1,000 simulations producing an outbreak of at least 1,000 people infected with a single introduction (Table 2) and increasing to 80% and 92% in the moderate and high transmission scenarios, respectively. On average, in the first 30 days of an outbreak following a single introduction, the estimated number of people infected ranges from 18 in the low transmission scenario to 370 in the high (Table 3). One year after the start of an outbreak, and in the absence of any effective interventions (e.g., vaccination, quarantine) or behavior change, between 73% (439,900/600,000; 95% prediction interval [PI], 62%–81%) and 98% (589,800/600,000, 95% PI, 96%–99%) of the population will have been infected (low and high transmission scenarios) (Table 2).

**Table 2:** Overall impact of simulated outbreaks in Kutupalong-Balukhali Expansion Site, assuming no effective novel interventions or behavior change. Total number of people infected, total hospitalizations, and total deaths represent the final cumulative counts after the simulated outbreak has run its full course, among simulations that resulted in outbreaks of at least 1,000 people infected. Maximum daily incident counts are the maximum incident counts of people infected and hospitalization on a single day across the full outbreak, occurring at the outbreak’s peak. We estimated the daily hospitalization capacity needed in beds using incident hospitalizations and assuming hospitalized individuals remain hospitalized for a mean of 11.5 days (95% CI, 8.0–17.3) [16]. We estimated the largest number of beds needed at any time over the course the outbreak, which we report as the maximum capacity needed. From the daily bed capacity needed over time, we also estimate the day on which this capacity needed exceeds the existing capacity of 340 beds, which we report as the day on which hospitalization need exceeds capacity.

Transmission scenario	Probability of outbreak of >1,000 people infected after a single introduction	Total number of people infected^†^ mean (95% PI)	Total hospitalizations of severe cases^†^ mean (95% PI)	Total deaths^†^ mean (95% PI)	Peak daily incident number of people infected^†^ mean (95% PI)	Peak daily incident hospitalizations^†^ mean (95% PI)	Peak hospitalization capacity needed (in beds)^†^ mean (95% PI)	Day on which hospitalization need exceeds capacity^†^ ^‡^ mean (95% PI)
**Low (*R***_***0***_ **= 1.5–2.0)**	61%	439,900 (378,200–484,700)	21,300 (17,100–26,600)	2,130 (1,700–2,660)	7,840 (5,060–10,470)	1,260 (690–2,290)	5,210 (3,120–8,090)	136 (96–196)
**Moderate (*R***_***0***_ **= 2.0–3.0)**	80%	546,800 (499,300–567,000)	26,500 (20,400–33,600)	2,650 (2,030–3,380)	17,200 (11,610–20,400)	2,400 (1,270–4,290)	10,240 (6,310–15,660)	81 (60–114)
**High (*R***_***0***_ **= 3.3–5.0)**	92%	589,800 (578,800–595,600)	28,800 (21,100–38,500)	2,880 (2,090–3,830)	28,480 (23,460–32,380)	3,640 (1,920–6,920)	15,450 (9,500–23,990)	55 (42–77)

†Among simulations with successful introductions that result in outbreaks.

‡ Current hospitalization capacity is estimated to be 340 beds.

Abbreviations: CI, confidence interval; PI, prediction interval

**Table 3:** Cumulative number of people infected, hospitalizations, ICU admissions, and deaths at 1, 3, and 12 months following successful introduction of simulations where an outbreak occurs.

Transm-ission scenario	1 month				3 months				12 months			
	*Number of people infected*	*Hospital-izations*	*ICU*	*Deaths*	*Number of people infected*	*Hospital-izations*	*ICU*	*Deaths*	*Number of people infected*	*Hospital-izations*	*ICU*	*Deaths*
***Low*** *(R*_*0*_ *= 1*.*5–2*.*0)*	18 (2–65)	1 (0–3)	0 (0–1)	0 (0–0)	2,127 (37–10,655)	71 (1–374)	6 (0–30)	3 (0–15)	421,500 (376,300–463,500)	20,500 (16,700–25,000)	6,500 (5,400–8,000)	2,040 (1,660–2,500)
***Moderate*** *(R*_*0*_ *= 2*.*0–3*.*0)*	54 (3–223)	1 (0–6)	0 (0–1)	0 (0–0)	125,838 (1,920–411,000)	4,000 (48–16,700)	183 (1–990)	123 (1–631)	546,800 (499,300–567,000)	26,500 (20,400–33,600)	8,500 (6,500–10,800)	2,650 (2,030–3,380)
***High*** *(R*_*0*_ *= 3*.*3–5*.*0)*	370 (4–1,850)	6 (0–28)	0 (0–1)	0 (0–1)	542,800 (260,300–594,300)	25,000 (5,900–37,200)	4,440 (98–10,100)	1,880 (94–3,440)	589,800 (578,800–595,600)	28,800 (21,100–38,500)	9,200 (6,700–12,300)	2,880 (2,090–3,830)

Abbreviation: ICU, intensive care unit

**Fig 1:**
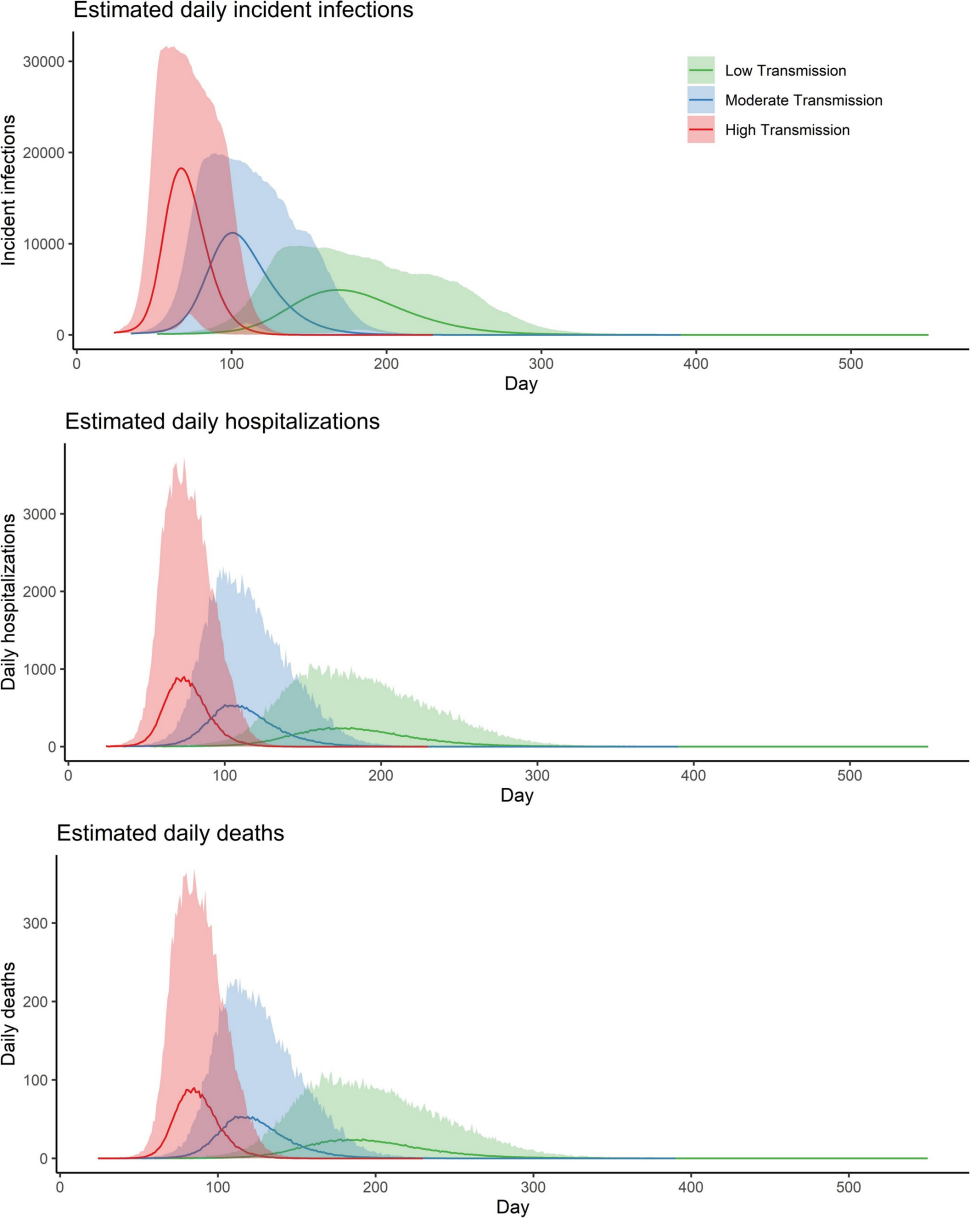
Simulated outbreak trajectories for Kutupalong-Balukhali Expansion Site camps under three transmission scenarios: low transmission (*R*_*0*_ = 1.5–2.0), moderate transmission (*R*_*0*_ = 2.0–3.0), and high transmission (*R*_*0*_ = 3.3–5.0). (A) Daily incident number of people infected by COVID-19, (B) daily incident hospitalizations, and (C) daily deaths under the three scenarios. The solid lines represent the mean outbreak trajectories, and the shading represents the 95% PIs of each scenario. PI, prediction interval.

In all scenarios, we observed relatively slow growth during the beginning of the simulated outbreaks (Fig 1), with limited numbers of people infected and few, if any, hospitalizations and deaths during the first month after the introduction of one infectious case (Table 3). However, this quickly changed once sufficient infections were in the population, with cases rapidly increasing and the outbreaks culminating within the year (Table 3, Fig 1). By the time the first hospitalization occurs, we expect 50 (95% PI, 1–197) individuals to be infected in the population under the low scenario, assuming homogeneous probability of infection by age. This increases to 72 (95% PI, 2–289) and 141 (95% PI, 3–502) in the moderate and high scenarios, respectively. When the first hospitalization occurs, we expect the virus to have been circulating in this population for an average of 38, 30, and 23 days under the low, moderate, and high transmission scenarios, respectively.

**Fig 2:**
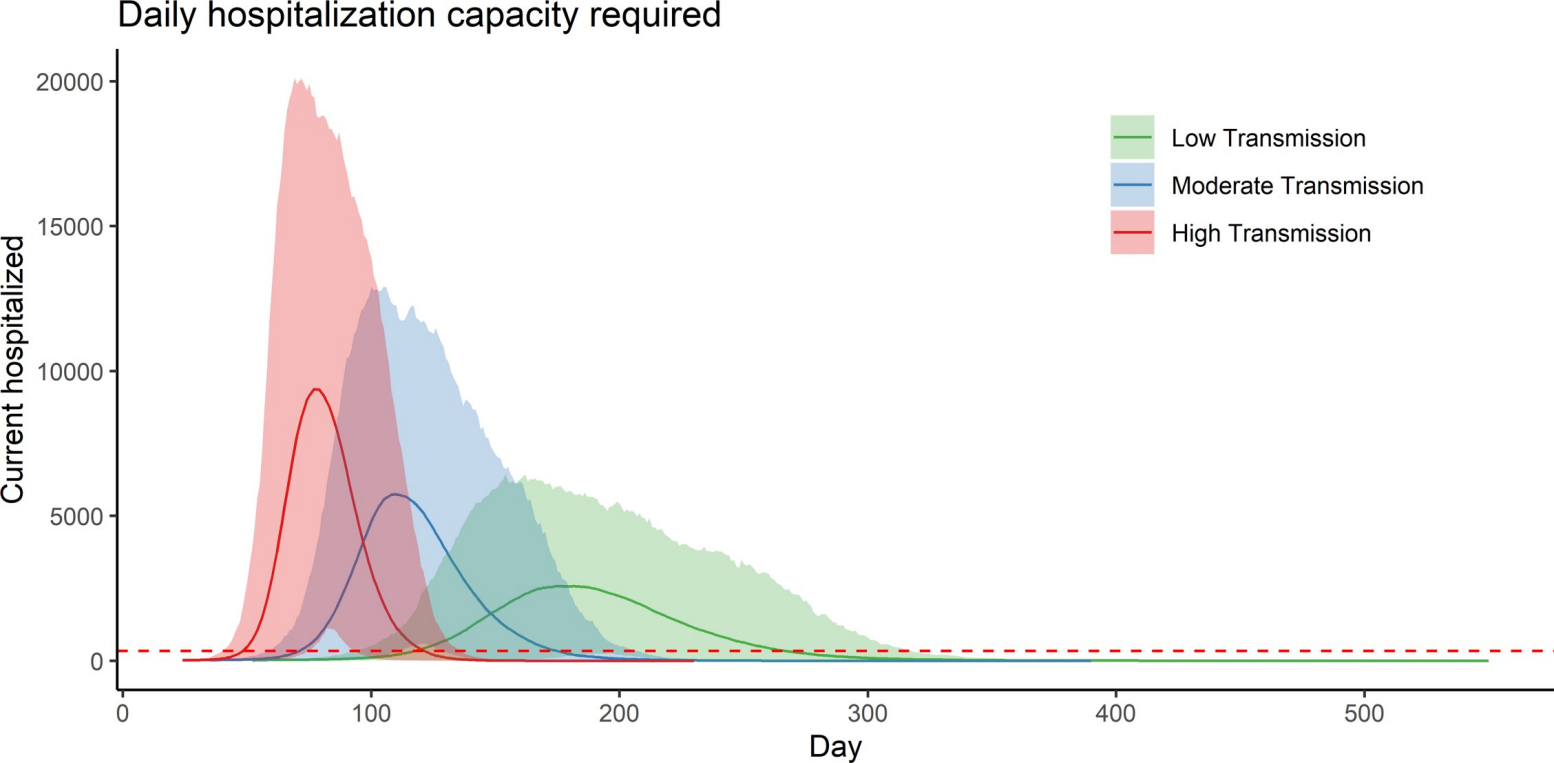
Hospitalization capacity requirements for an outbreak of SARS-CoV-2 in the Kutupalong-Balukhali camps, under three transmission scenarios: low transmission (*R*_*0*_ = 1.5–2.0), moderate transmission (*R*_*0*_ = 2.0–3.0), and high transmission (*R*_*0*_ = 3.3–5.0). The solid lines represent the mean outbreak trajectories and the shading represents the 95% PIs of each scenario. The dashed red line represents the 340-bed surge capacity currently believed to exist in the population. PI, prediction interval; SARS-CoV-2, severe acute respiratory syndrome coronavirus 2.

Adjusted for the age distribution in the Kutupalong-Balukhali Expansion Site, we estimated that 4.8% (95% PI, 0.3%–15%) of infections in this population would result in severe disease and hospitalization. The maximum daily hospitalization capacity needed ranges between 5,210 (95% PI, 3,120–8,090) in the low transmission scenario and 15,450 (95% PI, 9,500–23,990) beds in the high (Table 2). Under the low transmission scenario, hospitalization needs exceeded the hospitalization capacity of 340 beds after 136 days (95% PI, 96–196 days), while in the high transmission scenario, this occurred after only 55 days (95% PI, 42–77 days; Table 2, Fig 2). Within 3 months of successful introduction, we estimated 6 (95% PI, 0–30) cumulative ICU admissions in the low transmission scenario compared to 4,400 (95% PI, 98–10,100) in the high transmission scenario (Table 3).

Assuming that 10% of severe cases result in death, which puts the infection fatality rate at 0.48% (95% PI, 0.03%–1.5%), we estimated between 2,130 (95% PI, 1,700–2,660) and 2,880 (95% PI, 2,090–3,830) deaths from the low to high transmission scenarios (Table 2). In the high transmission scenario, the maximum number of daily hospitalizations is reached on day 80, compared to day 117 and day 190 on average in the low and moderate scenarios, respectively (Fig 1). Current hospital bed surge capacity (630) as reported from the site may almost double the number of available beds, which would delay overwhelming the capacity by 3–10 days on average (S2 Table).

## Discussion

The Kutupalong-Balukhali Expansion Site has 23 congested settlements, with nearly 600,000 persons residing there. Despite numerous challenges to the health of the population, including diphtheria and measles outbreaks, the health response and the resilience of the Rohingya refugees has been strong. However, the situation remains precarious and a COVID-19 outbreak in these camps would overload an already fragile health system and poor baseline health status. Using a stochastic disease transmission model, we estimated the number of people infected, hospitalizations, and deaths across three transmission scenarios after a successful introduction of SARS-CoV-2 into the Kutupalong-Balukhali Expansion Site. The introduction of SARS-CoV-2 into the Kutupalong-Balukhali Expansion Site or any other large refugee or internally displaced persons (IDPs) camp or settlement is likely to have serious consequences and overwhelm existing health systems. Even when transmission rates were assumed to be similar to that of influenza (low scenario), the necessary hospitalization capacities far exceeded the available capacities for the refugees in the expansion site in most simulations. Recent experience with other infectious disease outbreaks shows that the transmission of SARS-CoV-2 will likely be more intense in contexts such as the Kutupalong-Balukhali Expansion Site than in out-of-camp settings because of high population density, inadequate water and soap to maintain hygiene, limited ability to isolate infected individuals, and large household sizes [19].

The younger demographic profile of the Rohingya camp population could reduce the overall rate of severe cases as compared with China or other upper-middle and high-income countries that have older populations. After adjusting for age, we estimate the overall severe disease rate among infections in this population (mean = 4.8%, [95% PI, 0.3%–15%]) to be approximately half what we estimated for China (mean = 10.1% [95% PI, 1.8%–32%]) using the same methods (S1 Text). However, it is likely that comorbidities such as malnutrition, concomitant diseases, and poor overall health status could cause more severe outcomes among these groups, which was not captured by our model.

Existing inpatient facilities in the camps are already operating close to full bed occupancy, and all scenarios show that they will be overwhelmed. Under current conditions, we expect this to occur within 2 to 4.5 months (55 to 136 days) of the first introduction of the virus, with cumulative hospitalizations reaching 71 to 25,000 in the first 3 months, depending upon the scenario; these numbers rise dramatically by 12 months. Current hospital bed surge capacity as reported from the site may almost double the number of available beds, but even then, it only delays overwhelming the capacity by 3–10 days on average. Furthermore, while we have focused on the health needs of severe cases, health centers will be flooded with patients with mild to moderate symptoms also seeking care.

While we were unable to find an accurate recent estimate of the human resources currently available in the camps (e.g., number of doctors, nurses, midwives), it is extremely likely that they will be inadequate to face a large influx of patients. Given the limited availability of skilled health workers and hospital beds in Cox’s Bazar, it is unlikely that an additional influx of healthcare professionals within Bangladesh would be available. A surge of international healthcare workers could be considered, but this will be difficult given the high demands worldwide during the pandemic and existing travel restrictions. Furthermore, issues of introduction of the virus from expatriates into Bangladesh would need to be carefully addressed. A more likely strategy will be task shifting among existing healthcare workers, with physicians treating the most severe cases, nurses the less severe cases, and community health workers addressing mild infections. Such task shifting would require intensive training that needs to begin immediately. An examination of the “repurposing” of people working in various capacities (e.g., teachers as schools are closed) needs to be undertaken to assess how they can be used to address this epidemic while ensuring integration and complementarity of the response. Prepositioning of personal protective equipment (PPE) and comprehensive training that is constantly reinforced to reduce healthcare worker exposures are critical to avoid sickness and death among this essential cadre. In particular, the community health workers will have to adapt their method of working to reduce chances of getting infected or infecting others. This may require using texts and calling people in the Rohingya dialect to ensure culturally appropriate communication and education. Furthermore, community health workers can help build trust among the Rohingya that contact tracing and isolation are necessary, and that they can have confidence in these services, given the long history of mistrust among the Rohingya and authorities in Rakhine state, Myanmar [32].

Given the limited number of beds for treating the predicted large number of severe COVID-19 cases in the expansion site, isolation through hospitalization, as is being done in other settings, will likely be very difficult. Alternative plans for the isolation of mild symptomatic infections are needed and are currently being discussed with the government of Bangladesh to help control such an outbreak, as was done during the 2017 diphtheria outbreak. Cholera treatment centers and diphtheria outbreak centers that are currently on standby are a low-hanging fruit for repurposing. Setting up inclusive and accessible temporary hospitals to triage mild cases in these populations will require significant support and coordination by the government, UN agencies, and NGOs and may be limited by physical availability of land. Therefore, detailed advanced planning of healthcare capacities, triage procedures, and isolation strategies should be finalized and shared widely as soon as possible to minimize the impact of an outbreak.

Novel and previously untried strategies for social distancing and quarantine will likely be necessary to control transmission in this setting. While culturally difficult and requiring strong socialization, isolating people over 60 years of age and those designated medically vulnerable together, so-called shielding, could be considered. One possibility could be isolating such groups among a group of families and relatives who have confidence in one another [33]. Consistent monitoring of fever and other symptoms combined with appropriate testing will be an integral part of this strategy. Any such shielding must be decided upon wholly by the community after much discussion. It would require sufficient resources to provide comprehensive care (e.g., food and supportive services) for the isolated community members. Because physical distancing will be extremely difficult, the use of face masks among those most at risk, and possibly the whole population, could be considered if there are eventually sufficient supplies. Rapid and creative solutions for improving hand hygiene, such as the installation of handwashing stations and distribution of hand sanitizer, combined with communication campaigns, will be needed. Access to accurate and consistent health information will be critical, as already rumors and misinformation have spread among Rohingya refugees about COVID-19, partly due to camp-wide restrictions on the internet and telecommunications [4]. In the future, the use of people who have recovered from COVID-19 infections will need to be considered, after more data become available as to their ability to become reinfected and transmit to others, as was done with Ebola survivors [34]. The COVID-19–specific programs suggested above will require a strong surveillance system with adequate testing, case finding and isolation, as well as other supplies such as PPE. Such systems and supplies are not yet feasible in the camps but will be essential to address the epidemic in this setting.

The three scenarios show varying degrees of increased mortality. Mortality due to COVID-19 is dependent upon various factors, particularly access to hospitals with ICUs and ventilators. Currently, there is only one facility with few mechanical ventilators in the camps and one facility with fewer than 10 ICU beds and no ventilators. Therefore, it is likely that mortality rates due to COVID-19 will be significantly higher here than in settings where nationals or refugees have such access. Reportedly, the ICUs and ventilators are not functioning in the district hospital in Cox’s Bazar, and thus access to healthcare facilities to treat severe cases of COVID-19 may be equally challenging for nationals. All-cause mortality in the Kutupalong-Balukhali Expansion Site is currently comparable to estimates for Chittagong division and nationwide, and are below emergency levels. However, the scenarios show a significant increase in mortality, ranging from 2,040 deaths (3.4/1,000/year) to 2,880 deaths (4.8/1,000/year) at 12 months due to COVID-19 alone; this does not take into account expected deaths that would have occurred during this time, as well as increased deaths due to other illnesses that are untreated due to the outbreak.

As in other major epidemics where healthcare capacity and access to it is already limited, major outbreaks like this can easily disrupt an already precarious health system [35]. Diversion of these limited health resources from existing health services, including vaccination, obstetrical care, and emergency care, may cause an increase in mortality due to disease that could normally be treated by the health system; this occurred in the Ebola outbreak in West Africa, where more people died from malaria than Ebola, and in Eastern Democratic Republic of Congo, where more people died from measles than Ebola [36, 37]. Such an increase in non–COVID-19 mortality is particularly concerning with the upcoming monsoon season in Bangladesh.

There are several limitations to this study. We are using a mass-action model, which tends to overestimate the size of outbreaks because populations are generally not closed and well mixed. However, due to the population density and often closed nature of refugee camps, transmission in these settings has been shown to act more closely to theory. Additionally, the current evidence on the natural history and key epidemiological properties of COVID-19 reflects the interactions of the SARS-CoV-2 virus with non-displaced populations, and even in those populations, many of these parameters remain poorly defined. Population structure and population health can lead to a widely different burden of disease, with no modifications to the virus. With the particular demographic characteristics and health status of refugee populations, like this one in Cox’s Bazar, we need to be cautious when developing guidance based on previously estimated properties of SARS-CoV-2/COVID-19. Clinical surveillance, laboratory confirmation, and documentation are key to generating new evidence specific to this population and potentially generalizable to other refugee settings. Thus, we used the existing data that were available from documents and personal communications.

While we are focused here on the possible impact of SARS-CoV-2 on the Kutupalong-Balukhali Expansion Site, most of these findings are applicable to other refugee and IDP camp-like situations. While governments’ preparedness and response plans for COVID-19 may mention forcibly displaced populations, it is the details, or lack of, that must be examined. The Inter-Agency Standing Committee recently released interim guidance for scaling up the COVID-19 response in camps and camp-like settings that highlights inclusion, protection, and readiness; and has since developed preliminary multisectoral preparedness and response plans [38]. This guidance, which applies the WHO guidance on COVID-19 preparedness and response [39] to populations in humanitarian crises, should be acknowledged in government and humanitarian agency decision-making.

Clear and detailed COVID-19 plans that explicitly state how existing programs will be adapted according to their context should be developed and shared as soon as possible to allow for preparation. For example, different methods of food and fuel distribution will need to be undertaken that address social distancing issues. Potential consequences that isolation and other adaptive programming may cause, such as an increase in intimate partner violence, physical and sexual abuse among unaccompanied minors and other vulnerable groups, and mental health issues, should also be considered, and planned for in a concrete manner. Such adaptive programming will be an iterative process, as these are exceptional circumstances, and we will all have to learn by trying different approaches and then to document the results. A portal should be established by the United Nations High Commissioner for Refugees (UNHCR) and the global health cluster, led by WHO, to document how such adaptive programs for refugees and IDPs are being undertaken in different contexts. There will be a need for increased funding and basic supplies, including PPE as well as large-scale testing capabilities, if these plans are to succeed.

The scenarios presented in this study focus on camp-like settings, where refugee populations are relatively accessible and health services are available and generally free. However, the majority of refugees reside outside of camps, often in urban contexts [40]. For these populations, availability, access, cost, and quality of health services vary by host government policies. These refugee populations are also particularly vulnerable to the serious consequences of pandemics as targeted responses among a dispersed group of refugees, and equity in health service delivery is very difficult to achieve.

During exceptional times, it is not unreasonable for governments to take extraordinary measures to protect their citizens; in fact, that is what is expected of governments. Therefore, while difficult for governments to state openly at this point, it is possible that many countries will restrict access to their hospitals to nationals only, particularly when there are large numbers of refugees in the same geographical area. We are already seeing governments closing their border to nonnationals and, in some cases, not allowing people to seek asylum [41]. Such a predicament leaves refugees and other nonnationals, such as undocumented migrants, in an extremely precarious position. During a pandemic, there will be limited support by countries to establish field hospitals with ICU capabilities for refugees in camp-like settings, as they will be occupied with addressing their own national response. Unfortunately, there is no simple recommendation as to how to address this issue. However, we believe it is important to acknowledge the likelihood that host countries’ hospitals will be closed to refugees in most camp-like settings, and, consequently, COVID-19 preparedness and response plans should proceed based on realistic scenarios.

Finally, refugees face discrimination and are often falsely accused of spreading disease. The widespread rise of populism combined with anti-migrant and anti-refugee sentiments that we are observing globally provides a hostile environment that could be exacerbated by a pandemic. We are concerned that the COVID-19 pandemic, while completely unrelated to being a refugee, could be used as an excuse to take retribution against refugees, as well as other vulnerable groups such as IDPs and undocumented migrants. Such retribution could take many forms, including restricting or stopping asylum seekers, which is currently occurring, the closure of camps, and forced repatriation (refoulement, which is against refugee law), all used in the name of public health. Not only would this be morally wrong, it would jeopardize the effectiveness of containment and mitigation measures, as pandemics require planning and responses that do not discriminate by nationality, and protect the health of the global population.

Our findings suggest a COVID-19 epidemic among the Rohingya refugees in the Kutupalong-Balukhali Expansion Site will be extremely difficult to mitigate. Many of the current interventions such as social distancing, contact tracing and isolation, good hygiene, and sophisticated treatment in ICUs for critical cases will be challenging to implement. How SARS-CoV-2 will transmit and how COVID-19 will manifest in such a population, with a poor baseline heath status and acute malnutrition, is simply unknown at present. Beyond preparing now for COVID-19 through specific and sensitive interventions for this population to reduce morbidity and mortality, it is also important to rigorously document what does occur when the epidemic affects such a refugee population so we can learn how to better prepare and respond in similar settings. This will require strong surveillance systems to be established now. It will require working closely with the community to better understand how social distancing can work for them in their situation. And it will require the continued commitment of healthcare workers and the refugees themselves to respond to this pandemic.

## Supporting information

S1 Text Supplemental information. Additional details on the methods of the model and the severity age-adjustment.
(PDF)

S1 Table Comparison of selected indicators of health status and health service availability in the Kutupalong-Balukhali Extension Site and Bangladesh. (DOCX)

S2 Table Day on which hospitalization requirements exceed current estimated bed capacity (340 beds) and estimated surge capacity (630 beds) in the Kutupalong-Balukhali Expansion Site. (DOCX)

S1 Fig Bangladesh health facilities by type in Cox’s Bazar’s camp sites, Health Sector, Cox’s Bazar, March 2020. *Source*, *UN Office for the Coordination of Humanitarian Affairs;* *https*:*//www*.*humanitarianresponse*.*info/en/operations/bangladesh/health*
(TIF)

S2 Fig (A) Age distribution comparison between the Kutupalong-Balukhali Expansion Site, Bangladesh overall, and China and (B) the estimated probability of severe disease given infection with SARS-CoV-2, by 10-year age group. SARS-CoV-2, severe acute respiratory syndrome coronavirus 2.
(TIF)

S3 Fig Comparison of the expected overall proportion of infections that will result in severe disease, as estimated through adjustment for population age distribution. (TIF)

## Abbreviations

CI: confidence interval
ICU: intensive care unit
IDP: internally displaced person
NGO: nongovernmental organization
PHC: primary healthcare center
PI: prediction interval
PPE: personal protective equipment
SARS-CoV-2: severe acute respiratory syndrome coronavirus 2
SD: standard deviation
SEIR: Susceptible Exposed Infectious Recovered
UN WPP: United Nations World Population Prospects
UNHCR: United Nations High Commissioner for Refugees

## References

[1] United Nations High Commissioner for Refugees. Bangladesh: Operational Update. 2019.

[2] United Nations High Commissioner for Refugees. Country Profile: Rohingya Refugee Response Bangladesh. 2019.

[3] UNHCR Mapping Unit. Rohingya Refugee Emergency at a Glance 2018 [March 9, 2020]. Available from: https://www.arcgis.com/apps/Cascade/index.html?appid=5fdca0f47f1a46498002f39894fcd26f.

[4] ACAPS. COVID-19: Impact on the Rohingya Response. 2020.

[5] Strategic Executive Group. 2020 Joint Response Plan Rohingya Humanitarian Crisis. 2020.

[6] Emergency nutrition and health assessment, round 4, Cox’s Bazar, Bangladesh, September–October 2019, preliminary results.

[7] United Nations High Commissioner for Refugees. Dashboard—Public Health. 2019.

[8] MSF. Bangladesh Emergency Response Crises Info #9 –September 2018. 2018.

[9] World Health Organization. Rohingya Crisis in Cox’s Bazar, Bangladesh: Health Sector Bulletin. Bulletin. 2018;(05).

[10] World Health Organization, United Nations High Commissioner for Refugees, International Organization for Migration, Swiss Agency for Development and Cooperation, United Nations Population Fund. Available Services in Sadar Hospital, Ukhia and Teknaf Health Complexes. 2017.

[11] MSF. International Activity Report 2018 Bangladesh 2018 [March 9, 2020]. Available from: https://www.msf.org/international-activity-report-2018/bangladesh.

[12] Government of People's Republic of Bangladesh Ministry of Health and Family Welfare. Facility Registry: Cox's Bazar 250 Bed District Sadar Hospital 2020 [March 9, 2020]. Available from: http://facilityregistry.dghs.gov.bd/org_profile.php?org_code=10000922.

[13] Global Health Workforce Alliance. Bangladesh: World Health Organization; 2011 [March 9, 2020]. Available from: https://www.who.int/workforcealliance/countries/bgd/en/.

[14] World Health Organization. Health workforce requirements for universal health coverage and the Sustainable Development Goals.(Human Resources for Health Observer, 17). 2016.

[15] Bjørnstad O. SEIR model. McMaster University 2005 p. 1–8.

[16] Sanche S, Lin YT, Xu C, Romero-Severson E, Hengartner NW, Ke R. The Novel Coronavirus, 2019-nCoV, is Highly Contagious and More Infectious Than Initially Estimated. arXiv preprint arXiv:200203268. 2020.

[17] White LF, Wallinga J, Finelli L, Reed C, Riley S, Lipsitch M, et al Estimation of the reproductive number and the serial interval in early phase of the 2009 influenza A/H1N1 pandemic in the USA. Influenza and Other Respiratory Viruses. 2009;3(6):267–76. 10.1111/j.1750-2659.2009.00106.x 19903209 PMC2782458

[18] Kucharski AJ, Russell TW, Diamond C, Liu Y, Edmunds J, Funk S, et al Early dynamics of transmission and control of COVID-19: a mathematical modelling study. medRxiv [preprint]. 2020. 10.1016/S1473-3099(20)30144-4 PMC7158569 32171059

[19] Truelove SA, Keegan LT, Moss WJ, Chaisson LH, Macher E, Azman AS, et al Clinical and Epidemiological Aspects of Diphtheria: A Systematic Review and Pooled Analysis. Clinical Infectious Diseases. 2019 10.1093/cid/ciz808 31425581 PMC7312233

[20] COVID-19 Parameter Estimates: Github; 2020 [March 9, 2020]. Available from: https://github.com/midas-network/COVID-19/tree/master/parameter_estimates/2019_novel_coronavirus.

[21] Bi Q, Wu Y, Mei S, Ye C, Zou X, Zhang Z, et al Epidemiology and Transmission of COVID-19 in Shenzhen China: Analysis of 391 cases and 1,286 of their close contacts. medRxiv [preprint] 2020. 10.1016/S1473-3099(20)30287-5 PMC7185944 32353347

[22] United Nations Population Division. World Population Prospects: 2019 Revision. United Nations New York, NY; 2019.

[23] Lauer SA, Grantz KH, Bi Q, Jones FK, Zheng Q, Meredith HR, et al The incubation period of coronavirus disease 2019 (COVID-19) from publicly reported confirmed cases: estimation and application. Annals of internal medicine. 2020. 10.7326/M20-0504 PMC7081172 32150748

[24] Huang C, Wang Y, Li X, Ren L, Zhao J, Hu Y, et al Clinical features of patients infected with 2019 novel coronavirus in Wuhan, China. The Lancet. 2020;395(10223):497–506. 10.1016/S0140-6736(20)30183-5. PMC7159299 31986264

[25] Zhang G, Hu C, Luo L, Fang F, Chen Y, Li J, et al Clinical features and outcomes of 221 patients with COVID-19 in Wuhan, China. medRxiv [preprint]. 2020:2020.03.02.20030452. 10.1101/2020.03.02.20030452 PMC7194884 32311650

[26] Ferguson N, Laydon D, Nedjati Gilani G, Imai N, Ainslie K, Baguelin M, et al Report 9: Impact of non-pharmaceutical interventions (NPIs) to reduce COVID19 mortality and healthcare demand. 2020.

[27] Russell TW, Hellewell J, Jarvis CI, Van-Zandvoort K, Abbott S, Ratnayake R, et al Estimating the infection and case fatality ratio for COVID-19 using age-adjusted data from the outbreak on the Diamond Princess cruise ship. medRxiv [preprint]. 2020. 10.2807/1560-7917.ES.2020.25.12.2000256 PMC7118348 32234121

[28] COVID CDC Reponse Team. Severe outcomes among patients with coronavirus disease 2019 (COVID-19)—United States, February 12–March 16, 2020. MMWR Morb Mortal Wkly Rep. 2020;69(12):343–6. 10.15585/mmwr.mm6912e2 32214079 PMC7725513

[29] Dong Y, Mo X, Hu Y, Qi X, Jiang F, Jiang Z, et al Epidemiology of COVID-19 Among Children in China. Pediatrics. 2020:e20200702 10.1542/peds.2020-0702 32179660

[30] World Health Organization. Report of the WHO-China Joint Mission on Coronavirus Disease 2019 (COVID-19). 2020.

[31] Mahase E. Coronavirus: covid-19 has killed more people than SARS and MERS combined, despite lower case fatality rate. British Medical Journal Publishing Group; 2020. 10.1136/bmj.m641 32071063

[32] Tay A, Riley A, Islam R, Welton-Mitchell C, Duchesne B, Waters V, et al The culture, mental health and psychosocial wellbeing of Rohingya refugees: a systematic review. Epidemiology and psychiatric sciences. 2019;28(5):489–94. 10.1017/S2045796019000192 31006421 PMC6998923

[33] Dahab M, Zandvoort Kv, Flasche S, Warsame A, Spiegel PB, Waldman RJ, et al COVID-19 control in low-income settings and displaced populations: what can realistically be done?2020 March 21, 2020 Available from: https://www.lshtm.ac.uk/newsevents/news/2020/covid-19-control-low-income-settings-and-displaced-populations-what-can. 10.1186/s13031-020-00296-8 PMC7393328 32754225

[34] Bond N, Sesay S, Kamara A, Bates J, Teigen J, Stowlow J, et al Ebola Survivor Corps: employing Ebola survivors as health educators and advocates in communities affected by Ebola in northern Sierra Leone. The Lancet Global Health. 2019;7:S48 10.1016/S2214-109X(19)30133-0

[35] Parmar PK, Jin RO, Walsh M, Scott J. Mortality in Rohingya refugee camps in Bangladesh: historical, social, and political context. Sexual and Reproductive Health Matters. 2019;27(2):39–49. 10.1080/26410397.2019.1610275 31533592 PMC7888086

[36] Parpia AS, Ndeffo-Mbah ML, Wenzel NS, Galvani AP. Effects of response to 2014–2015 ebola outbreak on deaths from malaria, HIV/AIDS, and tuberculosis, West Africa. Emerging Infectious Diseases. 2016;22(3):433–41. 10.3201/eid2203.150977 26886846 PMC4766886

[37] Mahase E. Measles: Democratic Republic of the Congo recorded over 6000 deaths last year. British Medical Journal Publishing Group; 2020. 10.1136/bmj.m57 31915128

[38] Inter-Agency Standing Committee. Scaling-up COVID-19 Outbreak Readiness and Response Operations in Humanitarian Situations Including Camps and Camp-Like Settings. 2020.

[39] World Health Organization. 2019 Novel Coronavirus (2019‐nCoV): Strategic Preparedness and Response Plan. 2020.

[40] UN Refugee Agency. Global Trends: Forced Displacement in 2018. 2019.

[41] Joint Statement on US-Mexico Joint Initiative to Combat the COVID-19 Pandemic [Internet]. 2020. Available from: https://www.dhs.gov/news/2020/03/20/joint-statement-us-mexico-joint-initiative-combat-covid-19-pandemic

