# Supplementary material for: The potential impact of COVID-19 in refugee camps in Bangladesh and beyond: A modeling study
Source: PLoS Med. 2020 Jun 16;17(6):e1003144. doi: 10.1371/journal.pmed.1003144 (PMC7297408; doi:10.1371/journal.pmed.1003144)
Supplement: S1 Text — Additional details on the methods of the model and the severity age-adjustment. (PDF) [file pmed.1003144.s001.pdf]

Supplemental Information

The Potential Impact of COVID-19 in Refugee Camps in Bangladesh and Beyond: a modeling study

Shaun A. Truelove, Orit Abrahim, Chiara Altare, Stephen A. Lauer, Andrew S. Azman, Paul Spiegel

May 2020

METHODS

SEIR Model

We used a stochachastic SEIR (*Susceptible, Exposed, Infected, Recovered*) model to simulate transmission in population. We assumed a population size of 600,000, similar to the estimated population of the camps that make up the Kutupalong-Balukhali Expansion Site in Cox's Bazar, Bangladesh. We chose to use only this population to limit assumptions needed about connectivity and mobility with other areas and to keep the modeled population as close to a closed population as possible. Furthermore the population size is relatively static and interaction with the broader population in Bangladesh is relatively limited. We assumed homogenous mixing and infection rates by age. Hospitalization, ICU admission, and death were estimated from the transmission model, and not explicitly included as states in the model (see *Severity* section below).

Transmissmission was defined by the R_0 and serial interval. Time from exposure to development of symptoms was defined by the incubation period (σ). To capture a Ehrlang-distributed infectious period, we used $k = 3$ compartments for the infecteds (I_1, I_2, I_3). We assume transitions between compartments occur according to **S3 Table**.

S3 Table. Model transitions and rate parameters used in the SEIR model.

Transition	Rate Parameter	Unit
$S \longrightarrow E$	$\beta = R_0 \cdot \gamma$	d^{-1}
$E \longrightarrow I_1$	$\sigma = 5.2^{-1}$	d^{-1}
$I_1 \longrightarrow I_2$	$\gamma_1 = \gamma \cdot k$	d^{-1}
$I_2 \longrightarrow I_3$	$\gamma_1 = \gamma \cdot k$	d^{-1}
$I_3 \longrightarrow R$	$\gamma_1 = \gamma \cdot k$	d^{-1}

Severity

In a setting such as a refugee setting we assumed hospitalization will be limited to those who most require it, specifically severe cases. We assumed all hospitalization capacity available will be shifted for responding to COVID-19 severe cases, and no hospitalization for purposes of isolation will be done. Under these assumptions, proportion of infections resulting in severe disease approximate the proportion that will require hospitalization.

Age is currently the best defined predictor of severity due to SARS-CoV-2 infection, with severity increasing exponentially with increasing age. To estimate the age-adjusted severity, we used data from a several studies of COVID-19 transmission and hospitalization [1]. With these data, we used the *mgcv* package in *R* to fit a logistic generalized additive model with a cubic spline for age and a random effect for study to estimate the probability of severe disease given infection and uncertainty bounds for each age group [6]. These methods and code are detailed as part of the *covidSeverity* R package (https://github.com/HopkinsIDD/covidSeverity).

Age distributions for countries were taken from the United Nations World Population Propects (https://population.un.org/wpp/) [8]. We fit a spline to the reported age distribution (https://data2.unhcr.org/en/situations/myanmar_refugees) for the Kutupalong-Balukhali Expansion Site to estimate 10-year age proportions [9]. We used this population age distribution to weight the overall probability distribution of severity (**S2 Fig**).

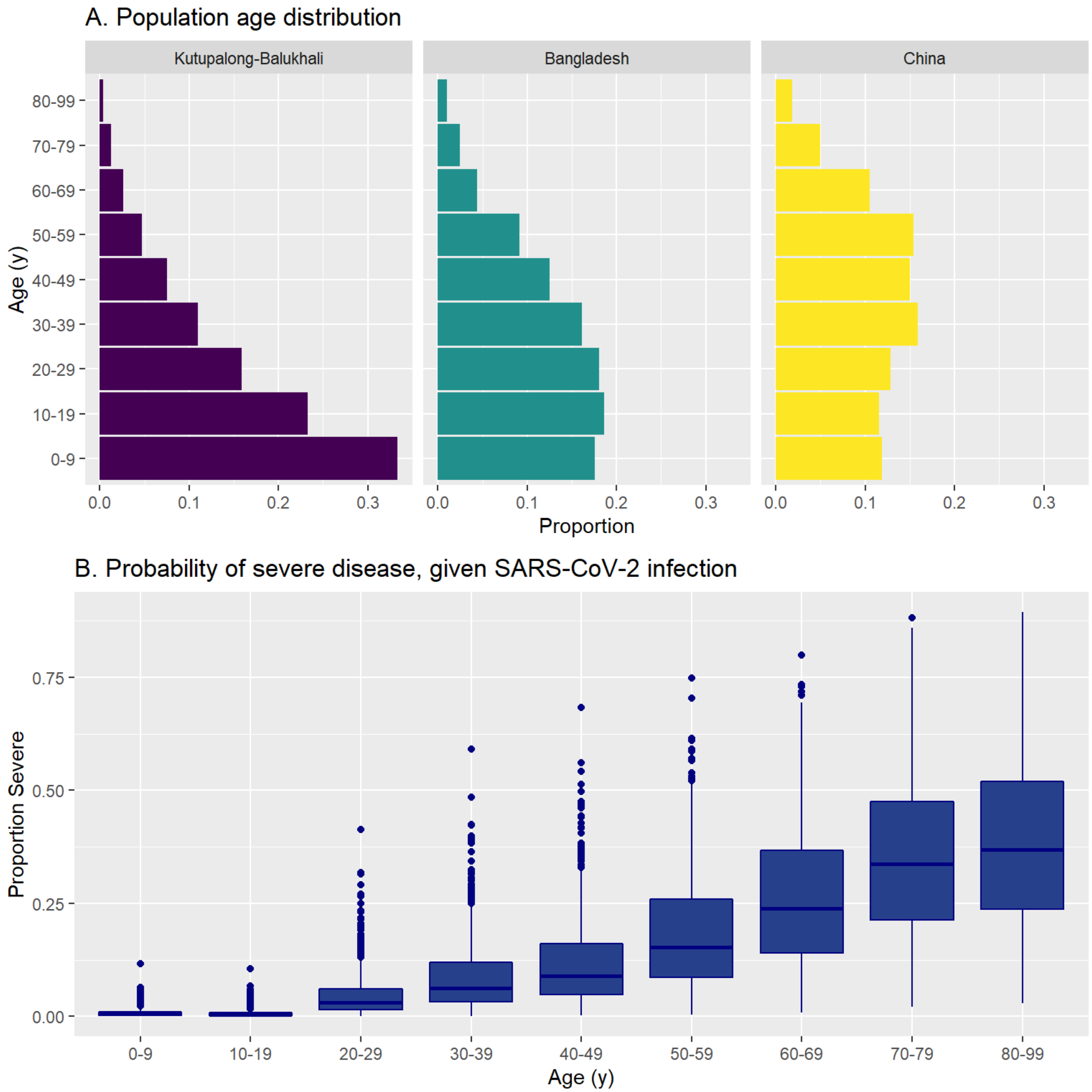

S2 Fig. (A) Age distribution comparison between Kutupalong-Balukhali Expansion Site, Bangladesh overall, and China, and (B) the estimated probability of severe disease given infection with SARS-CoV-2, by 10-year age group.

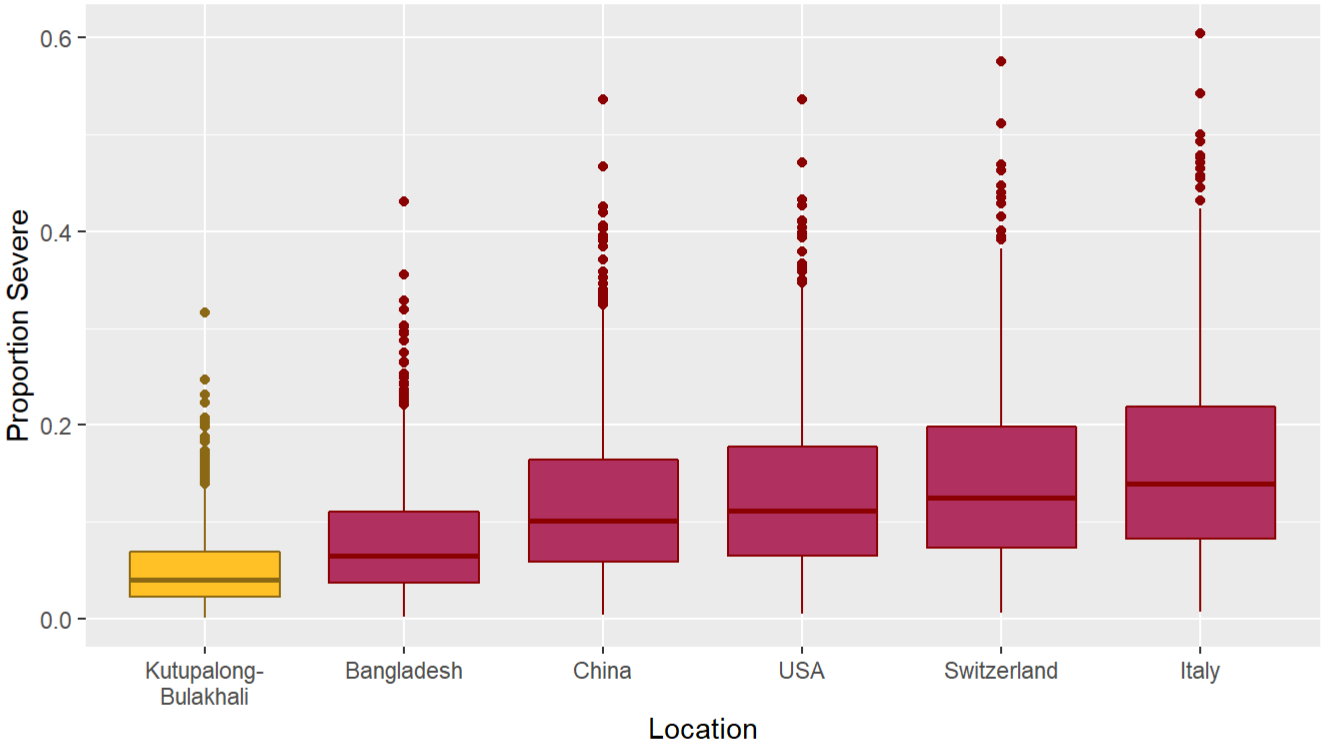

S3 Fig. Comparison of the expected overall proportion of infections that will result in severe disease, as estimated through adjustment for population age distribution.

References

1. Davies NG, Klepac P, Liu Y, Prem K, Jit M, CMMID COVID-19 working group, et al. Age-dependent effects in the transmission and control of COVID-19 epidemics. Epidemiology; 2020 Mar. doi:10.1101/2020.03.24.20043018 (https://doi.org/10.1101/2020.03.24.20043018)

2. Bi Q, Wu Y, Mei S, Ye C, Zou X, Zhang Z, et al. Epidemiology and Transmission of COVID-19 in Shenzhen China: Analysis of 391 cases and 1,286 of their close contacts. Infectious Diseases (except HIV/AIDS); 2020 Mar. doi:10.1101/2020.03.03.20028423 (https://doi.org/10.1101/2020.03.03.20028423)

3. Dong Y, Mo X, Hu Y, Qi X, Jiang F, Jiang Z, et al. Epidemiology of COVID-19 Among Children in China. Pediatrics. 2020 [cited 4 May 2020]. doi:10.1542/peds.2020-0702 (https://doi.org/10.1542/peds.2020-0702)

4. Ferguson N, Laydon D, Nedjati Gilani G, Imai N, Ainslie K, Baguelin M, et al. Report 9: Impact of non-pharmaceutical interventions (NPIs) to reduce COVID19 mortality and healthcare demand. Imperial College London; 2020 Mar. doi:10.25561/77482 (https://doi.org/10.25561/77482)

5. CDCMMWR. Severe Outcomes Among Patients with Coronavirus Disease 2019 (COVID-19) — United States, February 12–March 16, 2020. MMWR Morbidity and Mortality Weekly Report. 2020;69. doi:10.15585/mmwr.mm6912e2 (https://doi.org/10.15585/mmwr.mm6912e2)

6. Wood S. Mgcv: Mixed GAM Computation Vehicle with Automatic Smoothness Estimation. 2019. Available: https://CRAN.R-project.org/package=mgcv (https://CRAN.R-project.org/package=mgcv)

7. R: The R Project for Statistical Computing. Available: https://www.r-project.org/ (https://www.r-project.org/)

8. World Population Prospects - Population Division - United Nations. Available: https://population.un.org/wpp/ (https://population.un.org/wpp/)

9. Situation Refugee Response in Bangladesh. Available: https://data2.unhcr.org/en/situations/myanmar_refugees (https://data2.unhcr.org/en/situations/myanmar_refugees)
