# Supplementary material for: The potential impact of COVID-19 in refugee camps in Bangladesh and beyond: A modeling study
Source: PLoS Med. 2020 Jun 16;17(6):e1003144. doi: 10.1371/journal.pmed.1003144 (PMC7297408; doi:10.1371/journal.pmed.1003144)
Supplement: S1 Table — (DOCX) [file pmed.1003144.s002.docx]

**S1 Table**. Comparison of selected indicators of health status and health service availability in Kutupalong-Balukhali extension site and Bangladesh.

|  | **Comparison** | | **Standards** | |
| --- | --- | --- | --- | --- |
| **Indicators** | **Kutupalong-Balukhali  Extension Site (KB)** | **Bangladesh:**  **National (N),**  **Chittagong Division (C), and**  **Cox’s Bazar (CB)** | **WHO standards/**  **recommendations** | **Emergency Minimum**  **Standards^20^** |
| **Crude mortality rate (CMR)** ^a^ | KC ^b^ : 0.12/10,000/da y^4^  MS ^c^: 0.2/10,000/day | 0.13/10,000/day  0.14/10,000/day | *Does not exist* | 1/10,000/day |
| *(same CMR value converted to per population per year for*  *ease of comparison)* | KC: 4.32/1,000/year  MS: 7.2/1,000/year | 4.7/1,000/year (C) ^21^  5.1/1,000/year (N) ^21^ |  | 36.5/1,000/year |
| **Number of skilled health workers (doctors, nurses/midwives)** per 10,000 population | Not available | 6.3 registered physicians ^22^ | 44.5 ^13^,^d^ | 23 |
| **Community health workers**  per 10,000 population | 16.8 ^5,e^ | 2.13 (N) ^22^ | *Does not exist* | 10 - 20 |
| **Primary Health Care facility**  per population | 1 per 25,000 ^6^ | 1 per 39,394 (CB) ^23^ | *Does not exist* | 1 per 10,000  1 per 50,000 (rural) |
| **Hospital beds**  per 10,000 population | 5.7 [340 beds] ^24,f^  10.6 [630 beds surge capacity] ^g^ | 8 (N) ^25^ | *Does not exist* | 18 ^h^ |
| **District/ rural/ referral hospital**  per population and number of hospitals | Secondary hospitals:   - 1 per 119,200 [5 in KB site] ^24^   Tertiary hospitals: None available | Secondary hospitals (C)(N):   - 1 per 320,000 [89 in division] ^26^ - 1 per 386,000 [421 nationwide] ^26^   Tertiary hospitals (C) (N):   - 1 per 2,500,000 [11 in division] ^26^ - 1 per 2,600,000 [62 nationwide] ^26^ | *Does not exist* | District/ rural hospital:  1 per 250,000 |
| **Number of liters of water**  per person per day | 17.9 l/p/d collected at HH level ^27^ | *Not available* | 50 – 100 ^28^ | 15 |

**Notes:**

^a^ Mortality rates in emergency settings are calculated per 10,000/day, while in stable settings, they are calculated per 1,000/year. We presented the same mortality rate in both measures for ease of comparison with Bangladesh estimates.

^b^ KC: Kutupalong Camp

^c^ Makeshift Settlements (MS) include the Kutupalong MS, Balukhali MS and Expansion Zones, (which are part of the Kutupalong–Balukhali Expansion Site together with KC) as well as Hakimpara, Jamtoli, Potibonia, Chakmarkul, Unchiprang, Shamlapur, Leda, Ali Khali, Jadimura Shamlapur, and Nayapara Expansion (that are not part of the Kutupalong-Balukhali Expansion Site).

^d^ An aggregated estimate of the skilled health worker density needed to achieve 80% coverage of 12 selected Sustainable Development Goals’ tracer indicators.

^e^ Ratio refers to CHWs in all camps and settlements in Cox’s Bazar and total Rohingya refugee population (856,000).

^f^  This includes all hospital beds: adult inpatient beds, obstetric beds, and pediatric beds.

^g^ Surge capacity refers to the maximum number of beds available to accommodate a sudden influx of patients.

^h^ Standard excludes maternity beds.
