# Supplementary material for: The potential impact of COVID-19 in refugee camps in Bangladesh and beyond: A modeling study
Source: PLoS Med. 2020 Jun 16;17(6):e1003144. doi: 10.1371/journal.pmed.1003144 (PMC7297408; doi:10.1371/journal.pmed.1003144)
Supplement: S2 Table — (DOCX) [file pmed.1003144.s003.docx]

**S2 Table.** Day on which hospitalization requirements exceed current estimated bed capacity (340 beds) and estimated surge capacity (630 beds) in the Kutupalong-Balukhali Expansion Site.

| **Transmission Scenario** | **Day on which Hospitalization Need Exceeds Capacity** | |
| --- | --- | --- |
|  | ***Standard Capacity,***  ***340 beds***  *mean (95% CI)* | ***Surge Capacity,***  ***630 beds***  *mean (95% CI)* |
| ***Low*** *(R_0_=1.5-2.0)* | 136 (96-196) | 146 (104-207) |
| ***Moderate*** *(R_0_=2.0-3.0)* | 81 (60-114) | 86 (65-121) |
| ***High*** *(R_0_=3.3-5.0)* | 55 (42-77) | 58 (44-80) |
