# Supplementary figures and images for: The potential impact of COVID-19 in refugee camps in Bangladesh and beyond: A modeling study
Source: PLoS Med. 2020 Jun 16;17(6):e1003144. doi: 10.1371/journal.pmed.1003144 (PMC7297408; doi:10.1371/journal.pmed.1003144)

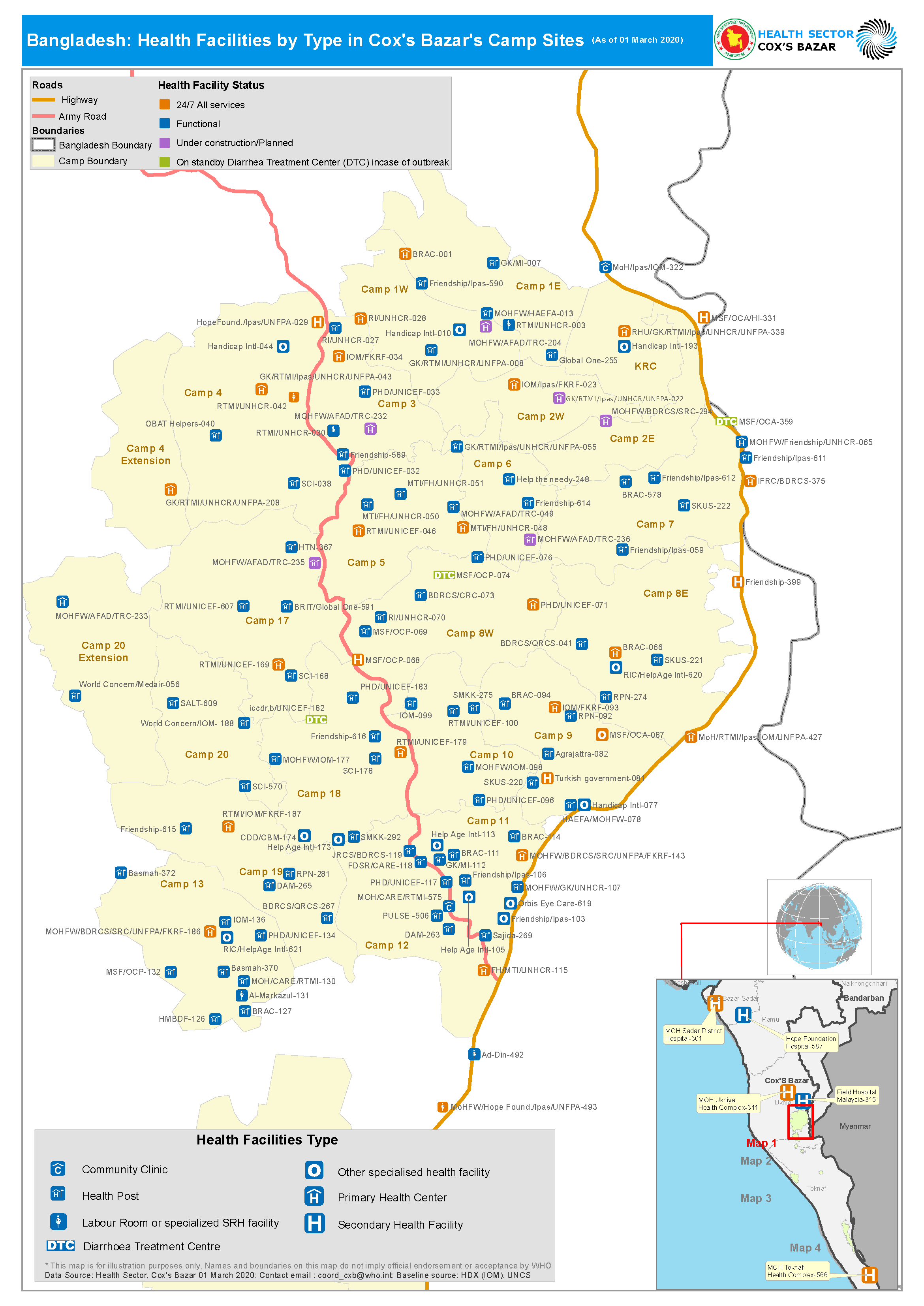

Supplement: S1 Fig — Source, UN Office for the Coordination of Humanitarian Affairs; https://www.humanitarianresponse.info/en/operations/bangladesh/health (TIF) [file pmed.1003144.s004.tif]
